# Integrating Gait Analysis Systems in Clinical Rehabilitation Settings for Individuals With Lower Limb Amputation: A Narrative Review

**DOI:** 10.7759/cureus.91030

**Published:** 2025-08-26

**Authors:** Xiaoning Yuan, Tawnee Sparling, David Carlisle, Shannon McGavin, Tiffany Riggleman, Emmanuel Cuevas, Shubham Ojha, Douglas G Smith, Riley Sheehan, Gabriel Kim

**Affiliations:** 1 Physical Medicine and Rehabilitation, Uniformed Services University of the Health Sciences, Bethesda, USA; 2 Physical Medicine and Rehabilitation, Walter Reed National Military Medical Center, Bethesda, USA; 3 phys, Uniformed Services University of the Health Sciences, Bethesda, USA; 4 Orthopaedics, William Beaumont Army Medical Center, El Paso, USA; 5 Orthopaedics, University of Washington School of Medicine, Seattle, USA; 6 Physical Medicine and Rehabilitation, Brian D. Allgood Army Community Hospital, Pyeongtaek, KOR

**Keywords:** clinical gait analysis, lower limb amputation, military, prosthetics, rehabilitation

## Abstract

A primary goal of rehabilitative care for individuals with lower limb amputation (LLA) is to restore safe, effective gait. Traditional gait analysis, both observational and quantitative, assesses gait deviations in individuals with LLA to inform clinical management. Observational gait analysis performed in clinical settings can be highly subjective, whereas quantitative gait analysis is often costly, time-consuming, and therefore impractical to perform in point-of-care clinical settings. To enhance clinical decision-making for individuals with LLA, it is beneficial to identify commercially available gait analysis systems that can be efficiently and effectively utilized in clinical settings. This narrative review presents categories of commercially available gait analysis systems, their capabilities, and prior implementation for gait analysis of individuals with LLA in clinical settings. Twenty-three articles of commercially available gait analysis systems (instrumented treadmills, instrumented walkways, inertial measurement unit-based, and markerless motion capture (MMC)) for the LLA population were included. This review of clinical applications of commercially available gait analysis systems identifies gaps in current clinical practice and offers recommendations to incorporate point-of-care gait analysis within the prosthetic phase of rehabilitation for individuals with LLA.

## Introduction and background

Globally, 57.7 million people live with acquired limb loss [1], with prevalence estimated to more than double from 2005 to 2050 in the United States alone [2]. Caring for individuals with lower limb amputation (LLA) requires interdisciplinary coordination among rehabilitation providers, including physiatrists, physical therapists (PTs), and prosthetists. A critical component of rehabilitation following LLA is prosthetic fitting and gait training. Despite concerted efforts by rehabilitation providers, individuals with LLA may exhibit persistent abnormal gait patterns, including markedly shortened step length, stiff-knee gait, and compensatory trunk lean [3]. Such gait deviations can result in inefficient ambulation and the development of musculoskeletal sequelae, such as low back pain [4] and osteoarthritis of the remaining joints [5]. Therefore, an important goal for individuals with LLA is optimizing their prosthetic fitting and gait training to enhance their overall functionality and quality of life, while mitigating the risks of secondary problems.

Serial evaluation of gait deviations is critical to inform clinical decision-making and guide prosthetic and rehabilitative care. Observational gait analysis (OGA) in clinical settings can be highly subjective and heavily relies upon clinicians’ visual interpretation of pathological gait determinants. OGA often focuses on the six determinants of gait: pelvic rotation, pelvic tilt, knee flexion in the stance phase, foot mechanisms, knee mechanisms, and lateral pelvic displacement [6]. While these provide a convenient framework, OGA is subjective, limited to descriptive documentation, and highly dependent on clinician experience and skill [7]. Alternatively, quantitative gait analysis (QGA) systems can provide more objective biomechanical data to optimize rehabilitation planning and prosthetic prescription [3,8,9]. “Gold standard” comprehensive QGA involves data collection via infrared camera-based three-dimensional (3D) motion capture systems, as patients walk along long, uninterrupted walkways (e.g., 16’×2’) with integrated force plates, and the use of wearable sensors. Such analyses can capture a wide range of variables (Table 1), including kinematic data, kinetic data, and temporospatial parameters.

**Table 1:** Definitions and examples of gait parameter outputs from the quantitative gait analysis. Authors' own creation

Gait Parameter	Definition [10]	Examples
Kinematic	Data on motion without regard to the forces that cause the motion	Joint angles in the sagittal, coronal, and transverse planes; trunk lean; pelvic motion
Kinetic	Data on motion with reference to the forces that cause the motion	Ground reaction forces; joint moments in the sagittal, coronal, and transverse planes
Temporospatial	Data of, relating to, or occurring in time and space	Velocity, cadence, step time, step length, stride length, step width, gait phase duration

Despite their benefits, gait analysis laboratories are costly, require significant space, and rely on highly trained staff to acquire and process data. A key challenge of comprehensive 3D quantitative gait analysis (QGA) is the need for time-intensive data processing, which can limit its availability for real-time, point-of-care decisions. Furthermore, the complexity of these reports may present a barrier to interpretation for some clinicians [10,11]. For example, the Department of Defense and Department of Veterans Affairs operate five state-of-the-art, fully equipped 3D gait analysis (3DGA) laboratories across the United States. These facilities represent a substantial investment, with construction costs exceeding $300,000 and annual maintenance costs of approximately $250,000 per laboratory. These costs cover real estate (e.g., space greater than 35' × 35' × 10'), specialized equipment (e.g., treadmills, high-performance computers, mounted infrared video cameras), custom construction (e.g., built-in force plates requiring a false floor), software (e.g., costs over $30,000, including maintenance), and the salaries of expert personnel (e.g., engineers, technicians, PTs, physicians) [12-14].

Given these time- and resource-intensive requirements, comprehensive 3D QGA is impractical to perform in many active clinical settings. Moreover, many service members and veterans who may benefit from 3DGA live far from these labs and encounter significant difficulty with access to this specialized care [12,15]. Commercially available, compact, and affordable gait analysis systems provide practical alternatives for the LLA population, as they may expedite quantitative and qualitative gait analysis. Although these systems may enhance the management of patients with LLA, pragmatic evaluation in clinical settings is essential. Optimally, these clinical 3DGA tools should 1) provide accurate, objective data output and analytics to support clinical decision-making and interventional management; 2) be performed quickly, efficiently, and reliably in clinical rehabilitation settings; and 3) serve as educational tools for patients and providers, including students and trainees. Proposed criteria for these systems include considerations of cost, ease of use (e.g., portability, space, and time requirements), clinically appropriate and useful data outputs (e.g., kinematic, kinetic, temporospatial), and prior validation and evaluation in the LLA population.

The objective of this narrative review is twofold. First, it provides a comprehensive overview of the main categories of commercially available gait analysis systems suitable for point-of-care clinical evaluation in the lower-limb amputation (LLA) population: instrumented treadmills, instrumented walkways, inertial measurement unit (IMU)-based motion capture systems, and markerless motion capture (MMC) systems. Second, it synthesizes the existing literature to identify gaps and propose recommendations for future pragmatic research and development aimed at optimizing point-of-care clinical gait analysis for this population.

## Review

Methods

The authors conducted a review of peer-reviewed literature reporting the use of commercially available gait analysis systems in clinical settings for the LLA population. Searches were conducted using PubMed and Google Scholar with a combination of primary keywords (“lower limb amputation”, “lower limb amputee”, or “lower limb loss” and “clinical gait analysis”) with secondary keywords (“instrumented treadmill”, “instrumented walkway”, “inertial measurement unit”, “markerless motion capture”). Relevant citations within eligible articles were also reviewed for inclusion. Inclusion criteria included full-length, peer-reviewed, English language manuscripts of commercially available gait analysis systems in clinical settings for individuals with LLA through January 2025. Exclusion criteria included case reports, conference abstracts, dissertations, and literature reviews. Selected articles of other populations with gait impairments were included, given the potential for technological translation to LLA. The following criteria were evaluated among available gait analysis options: cost, portability, time and space requirements, reliability and relevance of data outputs, and prior evaluation in the LLA population.

Results

A total of 740 articles were identified using PubMed and Google Scholar through January 2025. The authors screened the titles, abstracts, and/or full-length manuscripts against inclusion and exclusion criteria and for duplicates, with 11 articles meeting criteria. Using the references within the eligible articles, 12 additional articles were identified. In total, 23 publications met all criteria and were included in this review, summarized by system type.

Instrumented Treadmills

Instrumented treadmills rely upon integrated force plates to capture GRF, center of pressure (COP), and temporal (e.g., step time) measurements during gait [16]. Analysis typically requires data collection and processing by biomechanical engineers using specialized software and interpretation by trained clinicians. Force measurement capabilities vary depending on the type and design of the treadmill. Some are equipped with two parallel belts, each with its own force plate to collect data from each foot independently, while others report combined signals from both feet. Depending on the accuracy of COP measurements, estimates of spatial parameters (e.g., step length, step width) can be calculated. GRF and COP measurements provide the ability to identify subtle differences in gait symmetry, allowing instrumented treadmills to be used for gait evaluation in different clinical populations.

To date, instrumented treadmills have been used in both research and clinical settings to evaluate gait in different patient populations, such as common lower extremity injuries (e.g., patellofemoral pain syndrome) [17-19]. By modifying standard treadmills to measure GRFs [20] or evaluating commercially available systems, studies have focused on their utility in evaluating biomechanics in individuals with LLA. One study assessed running biomechanics and stiffness of running-specific prostheses (RSP) in Paralympic sprinters with LLA using a Treadmetrix instrumented treadmill (Treadmetrix, Park City, UT) and found significant differences between sprinters with unilateral and bilateral TTAs, compared to able-bodied sprinters, for the majority of variables examined (e.g., leg compression, leg stiffness, vertical GRFs) [21]. Furthermore, among athletes with unilateral TTA, researchers detected changes in RSP stiffness corresponding to alterations in prosthetic model, height, and/or sagittal plane alignment [22]. Another study used a Bertec instrumented treadmill (Bertec Corporation, Columbus, OH) with a motion capture system (Vicon Motion Systems Ltd, Oxford, UK) to produce an open kinematic and kinetic dataset for 18 individuals with unilateral TFA walking at different speeds, with the intention to support provider understanding of biomechanical measures and facilitate future research [23].

 Overall, there has been limited evaluation of instrumented treadmills within clinical environments for the LLA population. Instrumented treadmills can be expensive and often require semi-permanent installation for accurate measurements, limiting their portability and practicality in clinical settings (Table 2). While instrumented treadmills do not measure kinematics, they capture reliable kinetic and temporospatial data.

**Table 2:** Selected gait analysis systems with prior evaluation among individuals with lower limb amputation. Abbreviation: IMU = inertial measurement unit Authors' own creation

Category	Example	Ease of use				Data outputs
		Equipment needed	Cost	Portability, space requirements	Reporting time	
Instrumented Treadmills	Treadmetrix (Treadmetrix, Park City, UT)	Treadmill system, data capture card, external computer, software	$100,000 range, depending on treadmill specifications	Limited portability (e.g., ~2000 lbs), requires space for treadmill (e.g., 62” width × 100” length × 20” height)	Built-in clinical assessments available; requires additional processing, cleaning, and/or trimming time for custom metrics	Temporospatial, kinetic
Instrumented Walkways	GAITRite (CIR Systems, Inc., Franklin, NJ)	Sensor mat, external computer, external webcam, software	$10,000-$50,000, depending on mat size and software specifications	Portable mat but sensitive equipment, better suited for a more permanent space/setup	3-5 minutes to reporting	Temporospatial, kinetic
	Zeno Walkway (ProtoKinetics LLC, Havertown, PA)					
IMU-Based Motion Capture	APDM Opal (APDM Wearable Technologies Inc., Portland, OR)	Sensors, access points, chargers, docking stations, external computer, sensor straps, software	$10,000-20,000	Portable, no specific space requirements	Built-in clinical assessments available, requires additional processing time for custom metrics and software implementation	Temporospatial, kinematic
	ReLOAD	Sensors, earbuds, knee sleeves, mobile application, shoulder strap for tablet, tablet, waist belt	Not yet commercially available			
Markerless Motion Capture	KinetiGait (Running Injury Clinic, Alberta, CA)	Depth camera, external computer, software, treadmill	~$5000	Portable camera and laptop, limited portability of treadmill, requires space for the treadmill	~3 minutes to results	Temporospatial, kinematic

Instrumented Walkways

Instrumented walkways analyze gait variations by utilizing pressure sensors embedded into a mat. Data are collected and analyzed on an external computer with specialized software that reports clinical parameters. Standard metrics include temporospatial parameters, which can be repeated to monitor changes in an individual’s gait following clinical interventions [24]. Commercially available systems include the GAITRite (CIR Systems, Inc., Franklin, NJ) and the Zeno Walkway (ProtoKinetics LLC, Havertown, PA), which are designed to be cost-effective and clinically accessible.

GAITRite is a portable instrumented walkway [25] that has demonstrated concurrent validity of temporospatial parameters when compared to an eight-camera motion capture system (Motion Analysis Corporation, Rohnert Park, CA) within a stroke population [26]. Using GAITrite, researchers have identified significant gait deviations in veterans with LLA, specifically in parameters such as step length symmetry, step rate, and single limb stance, and captured information on their speed, endurance, and standard mobility [27]. Asymmetry in step length and stance time was specifically associated with poor performance-based physical function among adults with unilateral LLA at least one year post-amputation [28]. In addition, no differences in temporospatial parameters were found between individuals with unilateral TTA for vascular versus non-vascular causes, after controlling for velocity, balance confidence, and time since amputation [29]. Finally, veterans with TFA were found to ambulate with greater temporal but not spatial asymmetry than those with TTA, as a result of their prosthetic leg spending a greater percentage of the gait cycle in swing phase [30].

The Zeno Walkway is a gait and balance analysis tool, consisting of a portable mat with a grid of 0.4” pressure sensors connected in series. The system can measure pressure, spatial, and temporal parameters (e.g., relative pressure, gait phase, gait cycle, COP) during tasks such as static standing, straight walking, and abrupt stopping, and offers standardized protocols (e.g., walking, turning, static balance) [31]. Data collected by PKMAS can be used to monitor the efficacy of clinical decision-making during rehabilitation, progression in PT, training with adaptive devices, and improvement or regression of gait. QGA data collected by the system yield objective data to quantify functional gait outcomes, test intervention efficacy, and educate patients. Although the system can be costly, it can be easily set up in nearly any clinical environment, and data acquisition and processing occur in less than five minutes (Table 2). The Zeno Walkway was used to identify differences in temporospatial parameters that influence walking speed for 43 individuals with unilateral TFA and 49 with unilateral TTA, who performed five trials of the 10-meter walk test (10MWT) [32]. Prosthetic terminal double limb support, single limb support, and stride width accounted for nearly two-thirds of the variance in walking speed of individuals with TTA, whereas intact swing duration accounted for nearly half of the variance in walking speed for those with TFA. Taken together, these findings highlight the temporospatial gait parameters that can be targeted to improve walking speed for individuals with unilateral LLA. This study team also captured the temporospatial parameters of a 180° turning task for 40 individuals with unilateral LLA (20 TFA, 20 TTA) [33]. They found differences in the temporospatial characteristics of turning, with greater dependence on the intact limb by level of amputation (TFA > TTA), regardless of turn direction. These findings provide evidence to support novel prosthetic design and PT interventions to facilitate turning and reduce reliance on the intact limb for individuals with LLA.

The Zeno Walkway was also used to evaluate three transfemoral socket interface designs [ischial ramus containment (standard of care), dynamic socket (DS), subischial] for 13 participants with unilateral TFA (≥1 year of prosthesis use, independent community ambulatory status) [34]. While there were no differences in velocity or cadence, the DS demonstrated significantly greater symmetry in swing, stance, and single support percentage. Within 60 days after data collection, seven of 13 participants changed socket interfaces from the standard of care, suggesting that small differences in temporospatial gait parameters may contribute to clinically meaningful differences in individual patient preferences.

These findings demonstrate the potential benefits of using an instrumented walkway for point-of-care clinical gait analysis. Given the abundance of data collected by these systems, their reporting functions must be streamlined to highlight the most relevant gait parameters for providers to make point-of-care clinical decisions (e.g., step length, step width, single/double stance times, GRFs, dynamic foot pressure measurements), including normative ranges for comparison, in an accessible format that maximizes efficiency and benefit for patient care. Overall, findings from prior studies suggest that instrumented walkways, which only capture pressure and temporospatial parameters, offer promising alternatives to 3D laboratory gait analysis and are viable options for use in clinical settings [24,25,27].

IMU-Based Motion Capture Systems

IMUs are wearable sensors that use accelerometers, gyroscopes, and magnetometers to measure acceleration, angular velocity, and orientation, respectively, to derive kinematic and temporospatial parameters for real-time analysis of gait [7,35]. Single or multiple IMUs can be utilized on different body regions to collect data, depending on calibration and parameters of interest [7]. Data processing from IMU collection can require significant time and technical expertise, which can hinder utilization in clinical settings. Not all IMU data are comparable, as variability exists among IMU-based algorithms in step detection and demarcation (e.g., final contact) [36,37], which impacts the quality and interpretation of temporospatial parameters [7,38]. In addition, IMU systems have their own specifications, which can complicate the use of different models simultaneously or interchangeably due to potential incompatibility issues. Limited studies have used IMUs to evaluate individuals with LLA in clinical settings, examining trunk, pelvic, bilateral thigh, and/or ankle IMU-based parameters indicative of gait stability or symmetry, and reporting validity and reliability of mean temporospatial [39] and sagittal kinematic parameters [37,40]. In a study of 15 individuals with LLA, IMUs (Physilog®4, Lausanne, Switzerland) were placed on the participants’ bilateral feet during a six-minute walk test (6MWT) at the completion of their rehabilitation programs. Researchers found variability in temporospatial and kinematic gait parameters (e.g., minimum toe clearance, speed, cadence, stance time, foot flat ratio) between intact and prosthetic feet increased during the 6MWT, concerning for greater risk of future mobility problems after community reintegration [41]. They suggested the quantitative data provided by IMUs during a 6MWT may serve to assess clinical rehabilitation programs and assist clinicians with refining therapeutic interventions.

IMU systems used in populations with LLA include the APDM Opal (APDM Wearable Technologies Inc., Portland, OR) and the Rehabilitative Lower Limb Orthopedic Accommodating Device (ReLOAD), which is not yet commercially available. APDM Opal IMUs are small wireless devices around the size of a wristwatch [42]. In a study of nine individuals with LLA, APDM Opal sensors were placed on shoes, sternum, lumbar spine, and wrists to assess the relationship between gait symmetry, based on temporospatial measures, and patient-reported and performance outcomes related to function and mobility. While achieving gait symmetry after LLA is a common rehabilitation goal, better balance and walking performance associated with greater gait symmetry did not translate to better patient-reported outcomes, suggesting that achievement of symmetry was not related to self-perception of functional mobility in this cohort. An Instrumented Stand-and-Walk Test developed by APDM Mobility Lab captures stability-related measures (e.g., sway, anticipatory postural adjustment (APA), trunk ROM, temporal variability, turning) among LLA prosthetic users. Clinical feasibility was achieved, as few additional resources and space were required to perform the 15-minute assessment, which PTs and prosthetists may elect to perform during initial or follow-up evaluations. However, the system failed to capture APA data for all participants with prosthetic knees, demonstrating only partial construct validity [43].

ReLOAD is an IMU-based system that assesses the walking quality of individuals with LLA, combining Multi-Axial Profile Recorder sensors with a comprehensive telerehabilitation program providing real-time auditory biofeedback on gait deviations for individuals with LLA [44]. Five wearable sensors are placed on the sacrum, shanks, and thighs of participants to capture temporospatial and kinematic parameters [45]. In a test-retest reliability study, 128 individuals with unilateral TFA or TTA were examined, performing the 10MWT while donning four IMUs (i.e., shanks and thighs), which reliably detected differences in segmental symmetry and repeatability measures between prosthetic and sound limbs. The researchers posited that the system may serve as an objective, quantitative tool in the clinic [46], together with OGA, that informs rehabilitative and prosthetic care. ReLOAD was also evaluated for its efficacy as a telerehabilitation intervention in a study of 17 high-functioning service members and veterans with unilateral LLA [47]. Participants presented with significant improvements in hip extensor strength, mobility, endurance, and gait quality after an 8-week walking and home exercise program. The majority of participants reported they would use ReLOAD again for telerehabilitation, and more than half felt the real-time auditory biofeedback improved their gait [48].

While research studies have shown that IMUs can serve as effective, portable, and relatively low-cost tools that guide the management of patients with gait deviations (Table 2), further implementation of IMUs within clinical settings for the LLA population is needed to fully evaluate their benefit in fitting and training individuals during the prosthetic phase of rehabilitation.

MMC Systems

MMC systems are fully automatic systems with the potential to improve the feasibility of 3DGA in clinical settings. These systems were previously implemented for sports and human performance, injury prevention, and neurological rehabilitation [49]. Marker-based motion capture systems, which track joint kinematics using wearable markers, require significant resources in terms of time, space, and personnel, often limiting their use in routine clinical settings [12-14]. Moreover, marker sets can be cumbersome and affect the natural movements of participants. In contrast, MMC systems implement artificial intelligence (AI) and machine learning for pattern recognition of anatomical landmarks to calculate joint kinematics using a camera-based system and pose estimation algorithm. MMC systems may reduce the time and personnel resources required for marker-based systems. However, algorithm optimization, data processing, and analysis using markerless methods still require specialist time and expertise, and little precedent exists for clinical implementation within the LLA population.

The accuracy of MMC systems is influenced by the camera type and pose estimation algorithm used to identify anatomic keypoints (e.g., joint center locations, hip, knee). Notably, algorithms will only be as accurate as their training data used. Camera types differ and can be employed as single or multi-camera systems (e.g., 2D or 3D single camera MMC, 3D multi-camera MMC). Standard video cameras capture a sequence of images, while depth cameras record both images and depth, reducing variation arising from shadows or imperfect lighting conditions. Soft tissue movement or artifact and clothing (e.g., wearing baggy rather than tight-fitting clothing) can impact joint center detection and affect subsequent temporospatial and kinematic outputs.

Depth camera-based systems have the potential to be effective clinical assessment tools that measure temporospatial and kinematic parameters [50] and identify gait asymmetries, even with a small number of gait cycles [51]. In the healthy population, studies have demonstrated good agreement between 3D laboratory and depth camera-based gait analysis methods. In joints above the ankle, a majority of clinical gait parameters (e.g., trunk movement, postural transition time, different gait speeds from short walks) demonstrated good to excellent absolute agreement and consistency, when comparing a depth camera-based system (Kinect V2) with a 3D optical motion tracking system (Vicon Motion Systems Ltd) [52]. A proof-of-concept study reported that a real-time, remote monitoring tool for clinicians to assess home-based rehabilitation exercises, utilizing features extracted by depth cameras (Kinect v2), was reliable in measuring participants’ performance and able to discriminate between healthy participants and patients with neurological and orthopaedic conditions [53].

To the authors’ knowledge, no commercially available MMC system has been specifically designed for individuals with LLA. Motion analysis for four men with TTA and six control participants was performed in a study comparing sagittal kinematics between marker (Vicon Motion Systems Ltd) and MMC (Theia Markerless, Inc., Kingston, CA) systems [54]. Results revealed well-matched knee movement between the marker and MMC systems, but difficulty tracking pelvic motion, systematic offsets for ankle kinematics, and misinterpretation of movement using markerless methods (e.g., ankle dorsiflexion detected for a passive prosthetic foot in swing). The study authors noted that future AI training should use images of individuals with lower limb prostheses to improve MMC analysis within this population.

MMC systems hold potential as objective clinical gait analysis tools, but further research and implementation are required to determine the feasibility of current markerless methods to detect small, incremental changes in gait for individuals with LLA and provide clinically meaningful information that informs their rehabilitative and prosthetic management.

Discussion

This narrative review evaluated available literature on clinical applications of commercially available gait analysis systems for individuals with LLA. While each system type can be useful for gait analysis, our findings suggest that no single system is capable of providing cost-effective, time-efficient, and comprehensive 3DGA data.

Instrumented treadmills have been used to evaluate gait patterns in multiple clinical populations and are the only systems discussed in this review that provide kinetic data, demonstrating sufficient reliability in GRF and COP measurements to identify subtle differences in gait symmetry following LLA. However, instrumented treadmills are among the costliest commercially available systems, are not portable, require dedicated space for deployment, and only capture force data. Therefore, they may not be ideal for point-of-care clinical gait analysis and may instead be most effective in research settings, when paired with other motion capture systems.

Instrumented walkways are less costly and have been utilized in several studies for individuals with LLA. The system is relatively portable; however, it requires a large, uninterrupted space for setup, depending on the mat size (e.g., 4’ x 20’), and care must be taken to avoid damage to the embedded sensors with repeated system setup and breakdown. Importantly, these systems are reliable and time-efficient for gait analysis, requiring little to no data cleaning or processing, and can therefore be easily interpreted by providers and communicated with patients. However, only pressure and temporospatial measures can be reported, and further refinement in data reporting is needed to highlight the most relevant gait parameters for ease of interpretation by providers. Instrumented walkways may offer the most clinic-friendly option for point-of-care gait analysis.

IMU-based systems are relatively inexpensive, highly portable, and can provide kinematic analysis of functional movements beyond gait alone, to monitor progress and set goals during rehabilitation for the LLA population. However, the majority of kinematic and temporospatial gait parameters are estimated using sensor data by complex algorithms. While many measures have demonstrated reasonable reliability compared to “gold standard” motion capture, few to date have been designed for or tested among individuals with LLA, which may limit their accuracy for clinical gait analysis in this population. In addition, these complex algorithms typically require data processing before clinician interpretation, which necessitates time and technical expertise. Technology such as the ReLOAD system demonstrates promise for the LLA population, but it is not yet commercially available.

MMC, specifically depth camera-based systems, is relatively novel and inexpensive, with potential for gait analysis of individuals with LLA. However, the level of accuracy and reliability needed for clinical gait analysis has not yet been achieved. Moreover, pattern recognition using AI and machine learning must be trained using images of patients with prostheses to improve clinical utility and reliability within the LLA population. The efficacy of MMC systems may be limited by their dependence on standardized models for comparison, and further implementation studies are needed to determine their sensitivity to detect gait changes in the LLA population.

This narrative review has several limitations. It is susceptible to selection bias, as a targeted search for the LLA population was supplemented with literature from other gait-impaired populations when necessary. The synthesis of findings is also subjective, relying on the authors' interpretation rather than a systematic methodology. Consequently, this review cannot draw definitive conclusions.

## Conclusions

As the LLA population grows within the military and beyond, commercially available gait analysis systems that can effectively and efficiently yield objective, quantitative analytics on gait deviations can significantly impact functional outcomes and quality of life for patients. To implement these systems clinically for the LLA population, future research should prioritize refinement of the most significant and relevant gait measures to capture, which may depend upon the goal (e.g., gait symmetry, fall reduction, functional levels, return to duty). Appreciation of the gait parameters essential to achieve specific patient goals and optimize outcomes can, in turn, inform the choice of system. In addition, the reliability of systems must be established, relative to the gold standard, among individuals with LLA, to determine if a system is sufficiently accurate and reliable to identify differences in prosthetic fitting, gait training, and clinical decision-making over time. Finally, future system improvements or developments, including user interfaces and reporting, must be designed with the support and consultation of end-users, including providers and patients. To achieve the ultimate goal of integration in clinical rehabilitation settings, a gait analysis system must be easy to use, easy to process data, and easy for clinicians to interpret and communicate relevant and impactful findings to their patients with LLA.
