# Correction: Integrating Gait Analysis Systems in Clinical Rehabilitation Settings for Individuals With Lower Limb Amputation: A Narrative Review

**DOI:** 10.7759/cureus.c279

**Published:** 2025-08-29

**Authors:** Xiaoning Yuan, Tawnee Sparling, David Carlisle, Shannon McGavin, Tiffany Riggleman, Emmanuel Cuevas, Shubham Ojha, Douglas G Smith, Riley Sheehan, Gabriel Kim

**Affiliations:** 1 Physical Medicine and Rehabilitation, Uniformed Services University of the Health Sciences, Bethesda, USA; 2 Physical Medicine and Rehabilitation, Walter Reed National Military Medical Center, Bethesda, USA; 3 phys, Uniformed Services University of the Health Sciences, Bethesda, USA; 4 Orthopaedics, William Beaumont Army Medical Center, El Paso, USA; 5 Orthopaedics, University of Washington School of Medicine, Seattle, USA; 6 Physical Medicine and Rehabilitation, Brian D. Allgood Army Community Hospital, Pyeongtaek, KOR

This article has been corrected to include Riley Sheehan as the ninth author. His guidance was critical to the review article's direction and success. The authors deeply regret this oversight.

